# Curcumin Mediated Attenuation of Carbofuran Induced Oxidative Stress in Rat Brain

**DOI:** 10.1155/2016/7637931

**Published:** 2016-04-24

**Authors:** Sunil Kumar Jaiswal, Ashish Sharma, Vivek Kumar Gupta, Rakesh Kumar Singh, Bechan Sharma

**Affiliations:** ^1^Department of Biochemistry, University of Allahabad, Allahabad 211002, India; ^2^Department of Chemistry, KLPG College, Naini, Allahabad 211008, India

## Abstract

The indiscriminate use of carbofuran to improve crop productivity causes adverse effects in nontargets including mammalian systems. The objective of this study was to evaluate carbofuran induced oxidative stress in rat brain stem and its attenuation by curcumin, a herbal product. Out of 6 groups of rats, 2 groups received two different doses of carbofuran, that is, 15 and 30% of LD_50_, respectively, for 30 days. Out of these, 2 groups receiving same doses of carbofuran were pretreated with curcumin (100 mg/kg body weight). The levels of antioxidants, TBARS, GSH, SOD, catalase, and GST were determined in rat brain stem. The 2 remaining groups served as placebo and curcumin treated, respectively. The data suggested that carbofuran at different doses caused significant alterations in the levels of TBARS and GSH in dose dependent manner. The TBARS and GSH contents were elevated. The activities of SOD, catalase, and GST were significantly inhibited at both doses of carbofuran. The ratio of P/A was also found to be sharply increased. The pretreatment of curcumin exhibited significant protection from carbofuran induced toxicity. The results suggested that carbofuran at sublethal doses was able to induce oxidative stress in rat brain which could be attenuated by curcumin.

## 1. Introduction

Carbofuran (2,3-dihydro-2,2-dimethyl-7-benzofuranyl methylcarbamate) is an organocarbamate pesticide commonly known as Furadan. It is widely used to enhance food production in agricultural practices and also for protection of industrial and household items against unwanted pests and insects [1]. Due to the indiscriminate and continuous application and lipophilic nature of carbofuran, its presence has been detected in different environmental components such as soil and water [2], as well as in the mammalian systems like maternal plasma, umbilical cord, and blood of African-American women and new-born babies, respectively [3]. Carbofuran enters the biological systems via ingestion of pesticide-contaminated food and water as well as accidental or occupational poisoning [4] and causes severe toxicity to brain, liver, heart, kidney, and muscles [5–8].

Though the underlying mechanism of toxicity of carbofuran relies on reversible inhibition of acetylcholinesterase (AChE) activity via carbamylation of its serine residue at the synaptic junctions [1], it has been experimentally proved that the oral administration of carbofuran may also induce oxidative stress, which in turn may exert neuronal injury in the rat brain. The application of plant products such as the aqueous extract of* Cynodon dactylon* and vitamin C may cause attenuation of carbofuran toxicity [9].

Under normal physiological conditions, generation of free radicals and concomitant damage in the cells and tissues can be controlled by the cellular antioxidant defense system which comprises enzymatic and nonenzymatic constituents [10]. The enzymatic antioxidants comprise superoxide dismutase (SOD), catalase (CAT), and glutathione-S-transferase (GST), whereas the nonenzymatic antioxidants include vitamins C and E as well as the reduced form of glutathione (GSH). Oxidative stress is the consequence of the imbalance between the reactive oxygen species (ROS) production and antioxidant defense system of the cells or tissues [11].

The antioxidant compounds overcome the toxicity of oxidative stress generated or enhanced by a number of conditions like nutritional imbalance, xenobiotics and their metabolic products, strenuous physical activities, and hereditary disorders [12–14]. The attenuation properties of antioxidants protect the key biomolecules such as DNA, proteins, and lipids by scavenging reactive oxygen species (ROS). The use of curcumin, a herbal plant product, as an antioxidant is relatively safe as its toxicity is not reported even up to 10 g/day [15]. The curcumin displays antioxidant properties due to the presence of phenolics and methoxy groups on the phenyl ring and 1,3-diketone groups. It acts as a strong antioxidant by neutralizing free radicals and by showing metal binding characteristics [16]. Curcumin has potential to cross blood brain barrier in mammalian systems and exerts protective effect [17].

Neurological disorders such as epileptic seizures, demyelization, and dementia have relation between the free radical generation and oxidative stress [18]. Brain utilizes high amount of oxygen and energy. Due to existence of poor antioxidant defense system in brain, it becomes more prone to the oxidative stress as compared to other organs of the body. During the oxidative stress, the demand of oxygen to brain is elevated and the ATP consumption becomes more than its production. This metabolic stress resulted in enhanced production of ROS which causes damage to the neuronal membrane [8, 9]. The herbal plant products from the past are frequently used to reduce the oxidative stress. However, the attenuation of carbofuran induced oxidative stress in rat brain by curcumin is not documented. The present study was therefore designed to evaluate the extent of generation of carbofuran induced oxidative stress and its amelioration by curcumin in the brain stem of rat. It is the first report of its kind suggesting attenuation of chronic carbofuran toxicity at sublethal doses in the brain stem of rat.

## 2. Materials and Methods

### 2.1. Reagents and Chemicals

The technical grade (99.6% pure) carbofuran (2,3-dihydro-2,2-dimethyl-7-benzofuranyl methylcarbamate) in powder form was a kind gift of Rallis India Limited, Bangalore, India. Edible groundnut oil was purchased from the local market. Pyrogallol, reduced glutathione (GSH), 1-chloro-2,4-dinitrobenzene (CDNB), acetylthiocholine-iodide (ATI), 3,5-dithionitrobenzoic acid (DTNB), and bovine serum albumin (BSA) were purchased from Sisco Research Laboratories Pvt. Ltd., Mumbai, India. Curcumin was purchased from Sigma-Aldrich Inc., USA. All other chemicals used in the study were of analytical grade.

### 2.2. Animals

Thirty male Wistar rats weighing 100 to 130 g and at the age of 6 to 7 weeks were purchased from Central Drug Research Institute (CDRI), Lucknow, India. The animals were acclimatized for one week at ambient temperature in polypropylene cages in the laboratory. Each cage contains five rats in the laboratory under ambient environmental conditions. They had free access to the pelleted food (Dayal Industries Limited, Lucknow, India) and water. The experimental procedure was designed according to the guidelines of Institutional Animal Ethical Committee of University of Allahabad.

### 2.3. Treatment of Animals with Carbofuran and Curcumin

Thirty male Wistar rats were divided into six groups, each containing five animals.

- *Control Group*. It received only 0.5 mL groundnut oil orally for 30 days at the interval of 24 h.
- *15% Carbofuran Group (15% CF)*. It received 15% of LD_50_ (1.2 mg carbofuran/kg body weight) in 0.5 mL ground nut oil orally for 30 days at the interval of 25 h.
- *30% Carbofuran Group (30% CF)*. It received 30% of LD_50_ (2.4 mg carbofuran/kg body weight) in 0.5 mL ground nut oil orally for 30 days at the interval of 24 h.
- *Curcumin Group (Cur)*. It received 100 mg kg^−1^ body weight curcumin in 0.5 mL ground nut oil orally for 30 days at the interval of 24 h.
- *Curcumin + 15% Carbofuran Group (Cur + 15% CF)*. 100 mg kg^−1^ body weight of curcumin was given orally just before 30 min of carbofuran (15% LD_50_), treatment for 30 days at each interval of 24 h.

At the end of the treatment, all animals were anaesthetized with mild chloroform and sacrificed.

### 2.4. Preparation of Tissue Homogenates for Activity Assay of Antioxidant Enzymes and Estimation of Biomolecules

The brain stem homogenate (10%, w/v) was prepared in ice cold 0.25 M sucrose solution and centrifuged at 9000 ×g for 30 min at 4°C. The supernatants were separated by gentle decantation of centrifuged homogenates of tissues and used for assay of antioxidant enzymes and estimations of the levels of certain biomolecules.

### 2.5. Estimation of TBARS Levels

Lipid peroxidation was measured in the cytosolic fraction of the brain stem of rat by following the method of Niehaus and Samuelsson and the results were expressed as nmol TBARS mg^−1^ protein using 1.56 × 10^5^ M^−1^ cm^−1^ extinction coefficient [19].

### 2.6. Estimation of the Activities of Antioxidant Enzymes

The activity of superoxide dismutase (SOD, E.C. 1.15.1.1) was measured by using the method of S. Marklund and G. Marklund [20]. It is a spectrophotometric measurement of optical density of colored complex involving pyrogallol autooxidation at 412 nm for 3 min at the interval of 30 sec with or without the enzyme protein. One unit of the enzyme activity was expressed as 50% inhibition of autooxidation of pyrogallol per min.

The catalase (CAT, E.C.1.11.1.6) activity was measured according to the method of Beers and Sizer by measuring the decrease in the absorbance for H_2_O_2_ consumption at 240 nm at the interval of 30 sec for 3 min [21]. One unit of CAT activity was defined as micromoles of H_2_O_2_ decomposed per min using molar extinction coefficient of H_2_O_2_ (43.6 M^−1^ cm^−1^).

The activity of glutathione-S-transferase (GST, E.C. 2.5.1.18) was measured according to the method of Habig et al. [22]. The change in absorbance was recorded spectrophotometrically at 340 nm for 3 min at the interval of 30 sec and the results were expressed as *μ*mol mL^−1^ min^−1^ mg^−1^ protein.

### 2.7. Determination of the Levels of Reduced Glutathione (GSH)

The GSH content in the brain stem of rat was determined by the method of Ellman et al. [23]. Briefly, the 250 *μ*L of deproteinized supernatant of the brain stem homogenate was mixed with 100 *μ*L of 6 mM DTNB, 300 *μ*L of 200 mM phosphate buffer (pH 8.0), and 50 *μ*L of 300 mM NaOH. The optical density of the reaction mixture was measured at 412 nm. All the values were expressed as *μ*g mg^−1^ protein.

### 2.8. Determination of Total Protein in the Brain

The protein content present in different samples was measured according to the method of Lowry et al. using BSA as a standard [24].

### 2.9. Calculation of Oxidative Stress Index

The oxidative stress index has been expressed in terms of the prooxidant (P)/antioxidant (A) ratio and was calculated by the following formula:

(1) Oxidative stress index=Levels of MDALevels of Activity of SOD+CAT⁡+ GST.

### 2.10. Statistical Analysis

Data are presented as mean ± standard deviation (±SD) using Graph Pad Prism version 5.01 for Windows, Graph Pad Software, San Diego, California, USA. Data were analyzed using one way analysis of variance (ANOVA). Different groups were compared using Bonferroni's Multiple Comparison Test and considered significant at *P* ≤ 0.05.

## 3. Results

**Figure 1:**
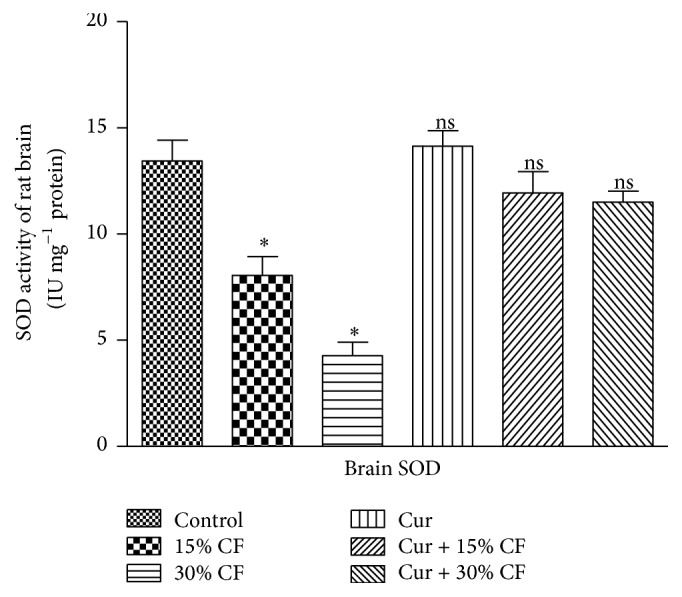
Effects of carbofuran and curcumin on the activity of SOD in the brain stem of rat. The procedures for the administration of carbofuran and pretreatment with curcumin as well as assay of SOD activity were the same as mentioned in Section 2. The unit of enzyme activity was expressed as IU mg^−1^ protein. The data represent mean ± SD of 5 independent experiments. *∗* indicates the *P* values significant at <0.001 and ns refers to nonsignificant at *P* > 0.05 as compared to control group.

**Figure 2:**
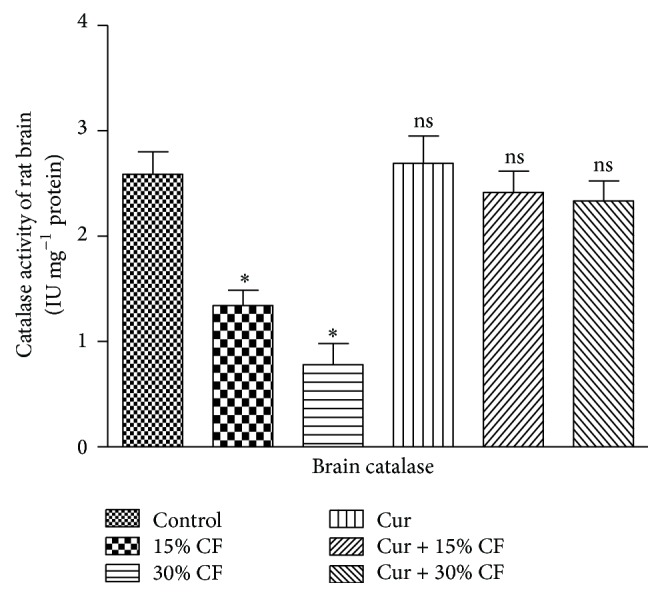
Effects of carbofuran and curcumin on the activity of catalase in the brain stem of rat. The procedures for the administration of carbofuran and pretreatment with curcumin as well as assay of catalase activity were the same as mentioned in Section 2. The unit of enzyme activity was expressed as IU mg^−1^ protein. The data represent mean ± SD of 5 independent experiments. *∗* indicates the *P* values significant at <0.001 and ns refers to nonsignificant at *P* > 0.05 as compared to control group.

**Figure 3:**
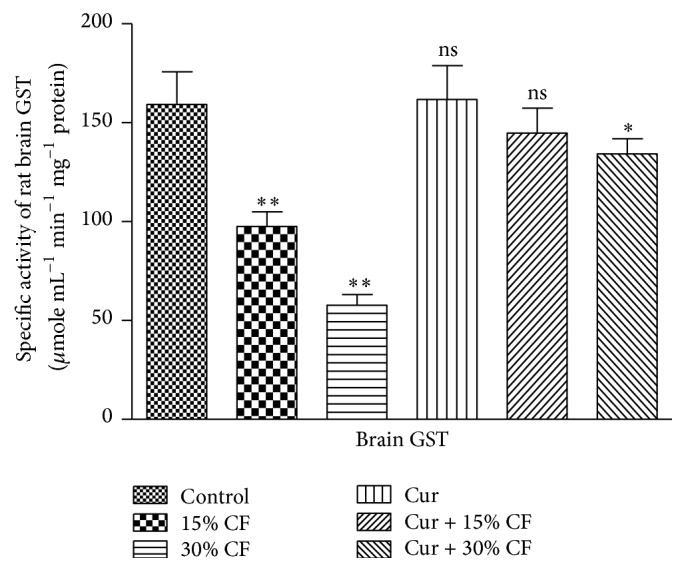
Effects of carbofuran and curcumin on the activity of GST in the brain stem of rat. The procedures for the administration of carbofuran and pretreatment with curcumin as well as assay of GST activity were the same as mentioned in Section 2. The unit of enzyme activity was expressed as *μ*mole mL^−1^ min^−1^ mg^−1^ protein. The data represent mean ± SD of 5 independent experiments. *∗* and *∗∗* indicate the *P* values significant at <0.001 and <0.05, respectively, and ns refers to nonsignificant at *P* > 0.05 as compared to control group.

The evaluation of the impact of carbofuran treatment at sublethal doses and the ameliorative effect of curcumin were monitored by determining the levels of TBARS and GSH in the rat brain tissues. The results presented in Table 1 indicated a significant (*P* < 0.001) increase in the level of TBARS and GSH in the brain stem tissue of rat by 57.2% and 84.25%, respectively, at 15% LD_50_ of carbofuran as compared to the control group of animals receiving only ground nut oil. The levels of TBARS and GSH in the brain stem of rat were further increased by doubling the dose of carbofuran, that is, 30% LD_50_, with the values being 102.27 and 218.6%, respectively, as compared to that of the control group. However, pretreatment of rats with curcumin resulted in significant recovery from the carbofuran mediated alterations in the levels of TBARS and GSH in the brain stem of rat. The pattern of recovery in the levels of TBARS and GSH was higher at lower dose of pesticide, and the low recovery was observed at higher dose of carbofuran (Table 1). The treatment of curcumin itself did not show any change in the levels of these indices even after treatment for 30 days. Since the exposure of rats to the two different sublethal doses of carbofuran displayed significant elevation in the levels of nonenzymatic indices (Table 1), the endeavours were made to examine the impact of same doses of carbofuran on the levels of enzymatic nonoxidants in the brain stem of rat for 30 days of treatment. The data concerning the enzymatic antioxidants such as SOD, catalase, and GST are presented in the Figures 1, 2, and 3, respectively. The exposure to 15% LD_50_ of carbofuran caused significant inhibition in the activity of SOD (Figure 1), catalase (Figure 2), and GST (Figure 3) to the tunes of 40.08, 48.18, and 38.69%, respectively, as compared to that of the control group. The significant inhibition in the activities of these antioxidant enzymes was further enhanced by 68.16, 69.8, and 63.63%, respectively, on doubling the dose of carbofuran (30% LD_50_). The pretreatment of curcumin followed by exposure of rats to carbofuran resulted in significant protection of the activities of SOD, catalase, and GST in the brain stem of rat (Figures 1–3).

The data obtained after calculation of oxidative stress index in terms of ratio of prooxidant (P)/antioxidant (A) in the rat brain are presented in Table 2. The values of P/A in brain were higher in the carbofuran treated animals and the trend of enhancement was dose dependent. However, prior treatment of curcumin in carbofuran treated animals displayed the value of P/A ratio near to that of control group.

## 4. Discussion

The oxidative process, such as lipid peroxidation of biomembranes, produces several compounds that are routinely used as molecular markers. Malonaldehyde is one of the most widely used indicators of the cellular redox state [25]. The results of the present study demonstrated that the brain stem of rat was under oxidative stress due to formation of reactive oxygen species (ROS) upon the exposure of sublethal doses of carbofuran; the extent of impact was dose dependent. The underlying mechanism of production of ROS might be due to direct effect of carbofuran or/and inhibition of cytochrome C oxidase [6]. Similar observation was also reported by Jaiswal et al. in rat brain at 5 and 10% LD_50_ of carbofuran [8]. The single i.p. administration of carbofuran in rat at different doses (0.2, 0.4, and 0.8 mg kg^−1^ body weight) also displayed similar effects in rat brain, which corroborates the finding from the present study [6]. In the present study, the level of TBARS was observed near to the control group when the carbofuran treated animals were pretreated with curcumin. The decrease in the level of TBARS might be due to the strong antioxidant property of curcumin. Curcumin may scavenge free radicals due to the presence of phenolics and methoxy groups on the phenyl ring and 1,3-diketone in this molecule [16].

Reduced glutathione (GSH) is a tripeptide acting as a natural nonenzymatic antioxidant. It can easily donate an electron. GSH is ubiquitous tripeptide involved in oxidation-reduction reactions, amino acid transport, detoxification of electrophile and metals, metabolites of xenobiotics, and many carcinogens and therefore it is considered as a biomarker of redox imbalance at cellular level [26]. In addition, it has a role in xenobiotic metabolism and is a first line of defense against oxidant-mediated cell injury [27]. The results from the present study reflected sharp increase in the level of GSH upon oral treatment of carbofuran; the extent of elevation was dose dependent. These results are in good agreement to that reported in the brain stem of rat and the heart upon long term exposure of animals to sublethal doses of carbofuran [7, 8]. The contrasting results have been reported in the liver of rat on acute exposure of carbofuran but the level of GSH was elevated in the liver upon chronic exposure of carbofuran [5]. The elevated level of GSH may be due to the activation of compensatory mechanism of animals treated with carbofuran. The pretreatment of curcumin caused significant restoration in the level of GSH in carbofuran treated animals in the present study. Similar observations have been recorded by Hemeida who reported the altered level of GSH in rat liver due to methotrexate and it was restored upon the curcumin administration [28].

Under normal physiological conditions, the enzymatic antioxidant defense system is best characterized by the levels of SOD, catalase, and GST as they are involved in alleviating the toxic effects of xenobiotics or their metabolic intermediates or end products by scavenging free radicals or reactive oxygen species (ROS). The toxic super oxides get converted into nontoxic molecular oxygen and H_2_O_2_ via dismutation. This reaction has been reported to catalyze by SOD. Further, H_2_O_2_ molecules get converted into nontoxic water and molecular oxygen by catalase. So any alteration occurring in the activities of SOD and catalase may cause serious pathophysiological alterations in the brain [29]. The significant reduction in the activities of these enzymes at both doses of carbofuran in rat brain (present investigation) showed the failure of antioxidant defense system due to the repeated oral treatment of carbofuran for 30 days. The similar observations were observed upon long term (30 days) exposure of experimental rats to relatively lower doses of carbofuran (5 and 10% LD_50_) in different body tissues of rats, such as brain and heart [7, 8]. However, similar observations were recorded in rat kidney when the animal was exposed to 1 mg kg^−1^ bodyweight up to 28 days [30]. The reduction in the activities of SOD and catalase was also reported in rat liver and kidney by another organocarbamate pesticide, methicarb, which may be responsible for generation of superoxide radicals [31, 32]. However, single dose administration of carbofuran has been reported to elevate the activities of SOD and catalase in rat brain and erythrocytes which may be associated with the adaptive mechanism of the antioxidant defense system [9, 11]. The results from the present study show activities of SOD and catalase near to control group animals on the prior treatment of curcumin in the carbofuran treated animals. This finding is supported by the reports from Nabavi et al., who indicated that the activities of these enzymes got restored by pretreatment of rats with curcumin near to control group in the cardiac tissues of sodium fluoride treated rats [33].

GST, like other two antioxidant enzymes as mentioned above, is also involved in maintaining a potent enzymatic defense system which protects the living cells against oxidative stress induced by xenobiotics [34]. The activity of this enzyme was significantly inhibited in rat brain as recorded in the present investigation upon exposure to sublethal doses of carbofuran when administrated orally for 30 days at the interval of 24 h. These observations are in agreement with those reported in erythrocytes and the brain of rats exposed to single dose of carbofuran for short duration, that is, 24 h [9, 11]. The results from a recent study have demonstrated that the rats exposed to relatively lower doses of carbofuran, that is, 5 and 10% LD_50_ for 30 days, exhibited significant reduction in GST activity in brain and heart [7, 8]. In contrast, GST from the liver of rat exposed to chronic and acute doses of carbofuran displayed elevation in its activity [5]. The activity of GST on the prior treatment of curcumin in carbofuran treated rat brain was restored near to control group animal which received only ground nut oil. This finding is supported by the observations recorded by Salama and El-Bahr [35].

The relation between LPO and antioxidant enzymes activities was best described by the prooxidant (P)/antioxidant (A) ratio as shown in Table 2. The ratio in the rat brain was increased in the carbofuran treated animals. The extent of enhancement in the ratio was dose dependent (Table 2). This trend showed that the free radical generation in the rat brain depends on the dose of carbofuran. The data indicated that brain is highly susceptible to oxidative stress due to the presence of the large amount of polyunsaturated fatty acids (PUFA), poor availability of antioxidants, and consumption of large amount of oxygen per unit weight (about 20% of the total amount used in humans) [6]. The prooxidant (P)/antioxidant (A) ratio was restored near to control group upon the pretreatment of curcumin in the experimental animals treated with carbofuran (Table 2). The restoration of this ratio was due to antioxidant potential of curcumin which restored the level of TBARS and activities of antioxidant enzymes.

## 5. Conclusions

These results from the present study demonstrated that carbofuran at sublethal doses was able to induce oxidative stress in the brain stem of rat, with the effect being dose dependent. It caused oxidative damage in the brain stem of rat upon long term exposure to carbofuran. The pretreatment of animals with curcumin showed attenuation of carbofuran toxicity. Its effect was more prominent at lower dose of carbofuran. The ameliorative effect of curcumin may be mediated via antioxidant potential of this molecule by scavenging the free radicals.

## Figures and Tables

**Table 1:** Effects of carbofuran and curcumin on the levels of MDA and GSH in the brain stem of rat.

	Control	15% CF	30% CF	Cur	Cur + 15% CF	Cur + 30% CF
MDA brain^a^	2.86 ± 0.27	4.49 ± 0.29^*∗*^ (+57.2%)	5.78 ± 0.60^*∗*^ (+102.27%)	2.73 ± 0.19 ns	3.07 ± 0.36 ns	3.27 ± 0.36 ns
GSH brain^b^	4.64 ± 0.73	8.56 ± 0.58^*∗*^ (+84.25%)	14.81 ± 0.78^*∗*^ (+218.6%)	4.36 ± 0.62 ns	5.50 ± 0.65 ns	6.12 ± 0.58 ns

^a^nmoles of MDA mg^−1^ protein; ^b^ *μ*g mg^−1^ protein; + sign shows increase in the MDA and GSH contents. The values are mean ± SD of five independent experiments. The procedure for estimations of MDA and GSH was the same as mentioned in Section 2. ns is nonsignificant as compared to control. *∗* indicates the *P* values significant at <0.001.

**Table 2:** Effects of carbofuran and curcumin on the oxidative stress index in the brain stem of rat expressed as the ratio of prooxidant (P)/antioxidants (A).

	Control	15% CF	30% CF	Cur	Cur + 15% CF	Cur + 30% CF
P/A	0.0163	0.0419	0.0930	0.0153	0.0192	0.0220

The oxidative stress index was calculated by determining the prooxidant (P)/antioxidant (A) ratio as shown in Section 2.
